# A Novel Two-Component System, Encoded by the s*co5282*/*sco5283* Genes, Affects *Streptomyces coelicolor* Morphology in Liquid Culture

**DOI:** 10.3389/fmicb.2019.01568

**Published:** 2019-07-09

**Authors:** Erick Eligio Arroyo-Pérez, Gabriela González-Cerón, Gloria Soberón-Chávez, Dimitris Georgellis, Luis Servín-González

**Affiliations:** ^1^Instituto de Investigaciones Biomédicas, Departamento de Biología Molecular y Biotecnología, Universidad Nacional Autónoma de México, Mexico City, Mexico; ^2^Instituto de Fisiología Celular, Departamento de Genética Molecular, Universidad Nacional Autónoma de México, Mexico City, Mexico

**Keywords:** *Streptomyces*, morphology, two-component systems, HAMP domain, aggregation, biofilm

## Abstract

*Streptomyces* are mycelial bacteria adapted to grow in soil. They have become important producers of biomolecules with medical applications, but their growth in industrial fermenters is challenged by their peculiar morphology in liquid culture: the hyphae tend to clump and grow as large pellets, which are oxygen- and nutrient-limited, grow slowly and present diminished protein production. Here, by implementing an experimental evolution strategy, a *S. coelicolor* strain, 2L12, with dispersed morphology and reduced pellet size in liquid culture and no defects in either differentiation or secondary metabolism was selected. Genome sequencing revealed a single amino acid substitution in a sensor kinase, Sco5282, of unknown function to be responsible for the morphological changes. Moreover, genetic and biochemical scrutiny identified Sco5283 as the cognate response regulator and demonstrated that the acquired mutation activates this two-component system. Finally, transcriptomic analysis of the mutant strain revealed changes in expression of genes involved in central processes such as glycolysis, gluconeogenesis, stress-signaling pathways, proteins secretion and cell envelope metabolism. Thus a novel two-component system is proposed to play a key role in the control of *Streptomyces* extracellular metabolism.

## Introduction

*Streptomyces* is a genus of soil-dwelling mycelial actinobacteria (Hopwood, 2007). In the presence of nutritious substrates in the soil, their spores are able to germinate, leading to growth of hyphae that adhere to the soil particles and to each other, and grow as a packed vegetative mycelium (Bobek et al., 2017). When conditions become suboptimal, they go through a developmental program, growing out of the soil and forming the reproductive aerial mycelium (Flärdh and Buttner, 2009). There is a concerted lysis of the vegetative hyphae, which releases nutrients for the growing aerial mycelium (Manteca et al., 2005). During this stage antibiotics are also produced preventing competitors from feeding on the lysed mycelium. Subsequently aerial hyphae septate by undergoing synchronous cell divisions, which later become chains of spores. This is the only stage of the *Streptomyces* life cycle in which unigenomic cells exist (Bush et al., 2015; Zhang et al., 2016).

This genus has been widely studied because of the vast array of antibiotics they produce. In recent years many species have also been exploited for the production of heterologous proteins. Because of their mycelial morphology, *Streptomyces* growth in liquid media is unlike that of unicellular bacteria. For example, germinating spores of the model organism *S. coelicolor* tend to clump together, and the hyphae stick to each other, leading to growth as tight mycelial pellets (Zacchetti et al., 2016). As a consequence, the inner hyphae have limited access to nutrients and oxygen (Walisko et al., 2015), leading to slower growth rates. This morphology in liquid cultures has also a clear impact on production of different molecules: while pelleted forms present increased antibiotic synthesis (Martin and Bushell, 1996; Manteca et al., 2008) they are disadvantageous for protein production, because of the reduced contact with inducer molecules (van Wezel et al., 2006).

For this reason, strategies have been developed to control the morphology of mycelia in liquid culture. Increasing agitation or viscosity of the medium (by addition of sucrose, for example) are simple methods that intensify shear forces, reducing aggregation but also increasing energy input and mechanical stress on the cells (van Dissel et al., 2014). A different approach has been to engineer strains where hyphae do not aggregate. For example, deletion of *cslA*, which encodes a cellulose synthase-like enzyme, leads to pellet dispersion. However, this mutant also exhibits pleiotropic effects on growth and differentiation (Xu et al., 2008). A different set of genes, *matAB*, were identified among the mutations in a dispersed-growing strain of *S. lividans*, a close relative of *S. coelicolor*, isolated from a chemostat after 100 generations (van Dissel et al., 2015). These genes were initially identified for their similarity to a biofilm operon of *Staphylococcus* spp., and their deletion was sufficient to induce dispersed growth in both *S. lividans* and *S. coelicolor* (van Dissel et al., 2015). More recently the *mat* genes have been shown to be responsible for synthesis of extracellular poly-β-1,6-*N*-acetylglucosamine, which provides hyphal adhesion to hydrophylic surfaces (van Dissel et al., 2018).

We sought to isolate new strains of *S. coelicolor* with a dispersed-growth phenotype through directed evolution. A study about the origin of multicellularity in the unicellular yeast *Saccharomyces cerevisiae* described an experimental evolution method to isolate aggregating mutants, based on the selection of fast-sedimenting cell clumps, eventually obtaining “multicellular” strains (Ratcliff et al., 2012). We adapted this method to obtain slower sedimenting *S. coelicolor* strains by applying the opposite selection: subculturing the slower sedimenting pellets. One of the obtained strains, 2L12, showed significantly reduced pellet size and dispersed growth even in medium without sucrose, although its secondary metabolism and differentiation in solid medium was not affected. We show that this phenotype is caused by an activating mutation in a histidine kinase of a novel two-component system. Finally, transcriptomic analysis of the 2L12 mutant strain revealed molecular targets able to explain the dispersed growth phenotype of the selected mutant strain.

## Materials and Methods

### *Streptomyces* Cultures

*Streptomyces* spores were obtained from MS agar plates and preserved in 20% glycerol at −20°C (Kieser et al., 2000). Liquid cultures were grown in LB + 25% sucrose or 2xYT (Kieser et al., 2000), without sucrose. Growth curves were performed in 2xYT or YEME media (Kieser et al., 2000), using freshly harvested spores to give an initial OD_450_ = 0.01. Samples (10 mL) were taken at the indicated times and filtered through 0.45 μm cellulose acetate filters (Millipore, HAWP02500); the mycelium was then dried at 100°C for dry weight determination. Total actinorhodin was determined by lysing 1 mL aliquots of the culture with 1 M KOH, which were then centrifuged to remove debris and measuring OD_640_ (Kieser et al., 2000).

### Experimental Evolution

Spores of *S. coelicolor* M145 were used to inoculate test tubes (16x150 mm) containing 2.5 mL of LB + 25% sucrose, which were incubated with orbital shaking at 30° C. After 48 h of growth, the tubes were left standing without shaking for 10 min to allow most of the mycelial pellets to sediment. Then, the upper 0.2 mL of each culture was transferred to a new tube with fresh medium and incubated as before. The process was repeated every 48 h until the culture consisted of slow sedimenting mycelium which appeared visibly dispersed. When this stage was reached, the mycelium was transferred to MS agar plates and allowed to sporulate. Spores were then streaked onto fresh plates for single colony strain purification. Spores from individual colonies were tested in LB + 25% sucrose, and spore preparations were made of those strains maintaining dispersed morphology after single colony purification.

### Microscopy and Pellet Size Determination

Six independent cultures of M145 and 2L12 were grown in non-viscous medium (2xYT), and the morphology of mycelial pellets was analyzed using a Nikon Eclipse 6000 microscope. In order to measure the complete pellets of strain M145, consecutive bright-field images under 10× amplification were taken and stitched together using the ImageJ plugin Stitching (Preibisch et al., 2009) as pellets of the wild type were often too large to fit in a single field. The area of the cross-section of the pellet was measured using ImageJ tools.

### DNA Extraction, Genome Sequencing and Mutation Analysis

For total DNA isolation, a modification of procedure 4 of Hopwood et al. (1985) was used. Briefly, 25 mL cultures of strains M145 and 2L12 were grown in LB+25% sucrose medium for 48 h, the mycelium was then harvested by centrifugation, washed with 25 mL of a 10% sucrose solution, and resuspended in 5 mL of lysis buffer (25 mM Tris–Hcl, 25 mM EDTA, pH = 8.0) containing 5 mg of freshly dissolved lysozyme and 250 μg of RNase A, and incubated at 37 C for 1 h. After lysis, 2.5 mL of a 2% SDS solution was added followed by vortexing for 30 s. The resulting lysed mycelium was extracted several times with an equal volume of saturated phenol:chloroform until a clear interphase was observed after centrifugation. Total DNA was then precipitated with ethanol, dried and resuspended in 0.5 mL of distilled water. The libraries were constructed with a TrueSeq DNA PCR-Free Library Prep kit (Illumina, 215962), and sequenced in an Illumina MiSeq equipment at a 2 × 150 cycles configuration at the Unidad Universitaria de Secuenciación Masiva y Bioinformática of the Universidad Nacional Autónoma de México^1^.

For variant calling, pair-end reads were aligned to the reference *S. coelicolor* A3(2) genome (NC_003888.3) using Bowtie 2 software (Langmead and Salzberg, 2012). Variant calling was performed using Samtools (Li, 2011) and VCFtools (Danecek et al., 2011). Only variants with a quality score above 100 were considered, and analyzed individually against the reference genome using Artemis (Carver et al., 2012). Variants present in both M145 and 2L12 were filtered out, as were variants with a lower quality score not present in at least 95% of all individual reads. The bam files used for variant calling, which contain all the sorted and aligned reads, are available from the NCBI with accession no. PRJNA499125.

### Sedimentation Time Determination

Spores of the strains to be tested were used to inoculate liquid LB + 25% sucrose medium to obtain dispersed mycelium, which was then used to inoculate 2xYT medium at a 1:20 ratio. Mycelial pellets were cultured for 48 h at 30°C. To measure the sedimentation time of the pellets, cultures were diluted with one volume of water and left to settle at the bottom of the tube before taking 0.5 mL to overlay on top of a 2 cm column of a 10% sucrose solution in a 13 × 100 mm test tube. The time reported in this work is the time it took for the first pellet in each tube to reach the bottom of the solution.

### Genetic Manipulation

PCR targeting (Datsenko and Wanner, 2000) was performed as described by Gust et al. (2004). The *aac3(IV)-oriT* (apra) cassette described by Gust et al. (2004), which confers resistance to apramycin, was amplified with oligonucleotides SCO5282For and SCO5282Rev (Table 1) and used to replace the *sco5282* gene in cosmid StCB12 (Redenbach et al., 1996). This cosmid (StCB12Δ*sco5282*::apra) was introduced by conjugation from the non-methylating *Escherichia coli* strain ET12567/pUZ8002 (Gust et al., 2004) into the wild type strain M145 to obtain apramycin-resistant exconjugants, which were then tested for kanamycin resistance. A kanamycin sensitive exconjugant was purified and confirmed by PCR as a Δ*sco5282*::apra null mutant (strain IB94). The StCB12Δ*sco5282*::apra cosmid was also used to clone the *sco5282-D125G* allele by introducing it into strain 2L12, and selecting exconjugants resistant to both apramycin and kanamycin, in order to obtain strains with the cosmid inserted into the chromosome. These strains were grown non-selectively in liquid culture, and covalently closed circular DNA was purified, which was then introduced into *E. coli* strain DH5α by transformation, with selection for kanamycin and ampicillin resistance. About half of the colonies were resistant to apramycin and carried the StCB12D*sco5282*::apra cosmid, whereas the other half were apramycin-sensitive and therefore carried the StCB12*sco5282-D125G* allele, which was confirmed by DNA sequencing. The StCB12*sco5282-D125G* cosmid was introduced into strain IB94 to replace the apramycin resistance cassette with the cloned *sco5282-D125G* allele, yielding strain M145*sco5282-D125G.* Allele replacement was confirmed by sequencing the PCR product obtained from chromosomal DNA of the resulting strain with primers SCO5282Up and SCO5282Down, which flank the kinase gene.

**TABLE 1:** Oligonucleotides used in this work.

**Name**	**Sequence 5′ → 3′**
SCO5282For	GCACGGCGTGGGCTACGCCTTGGAGACGCCGACGCC ATGATTCCGGGGATCCGTCGACC
SCO5282Rev	CGCAGTGGATCTTGACGCTCCGCTTCGAACCCTACG TCATGTAGGCTGGAGCTGCTTC
SCO5282Up	GTGGACAGCCACATCAAGG
SCO5282Down	GTTCATCGTCGCGTAAGTCA
SCO5283For	CCACGGATTCCGGAAAGCACACCTCAGGGGCGGGCG ATGATTCCGGGGATCCGTCGACC
SCO5283Up	GGATCATTCAAGCGTCAAGC
cl5282For	CATATGAGCAGCAGAGGACGGGA
cl5282Rev	GAATTCAACCGGACGTCGACGGCA
cl5282Sol	CATATGTCGCTGACCGCCCCGCTG
cl5283For	CATATGGAGCAGACACAGACCTCC
cl5283Rev	GAATTCATGGCGTCGGCGTCTCC
ATTB_FOR	CCATGCATGCACAGCTCAGGCAGACGTTA
ATTB_REV	GTGTGCATGCGACCGGTACTTGTCATGG
**For RT-qPCRs**	
0753for	GAAATGGCTATACGAAGGGAAG
0753rev	GACGGCGTTCACGTTCAG
0754for	ACCTCGAAGTCCACGAAGAG
0754rev	AGGGTCATGGAGGTCAGTTC
mbl2for	GGCATGATCCTCGACGTG
mbl2rev	TGGGTGTCGACGATGGAG
gap3for	CGATCGTCGAGCTGAACAC
gap3rev	GTGATGTCGGAGGAGACCAG
gap2for	GACGACTCGTTCACCAACTG
gap2rev	CTGACCACCGACTTGTTCAC
pckGfor	GAGGGCTTCTTCGTCAAGG
pckGrev	GCGAGATGTACTTGGTGCTG
whiDfor	AAGGAGGTCTGCATGAGGTG
whiDrev	CCCGTCCCATGAGTTCTTC
matBfor	ACGCCATGGACAACAAGTC
matBrev	TGTTGACGACGACGAGGTAG
sigBfor	GGGAGTTGTCGAAGCTGTTC
sigBrev	ATCTCGATCAGCGTGTTGC
hrdBfor	GGTCGAGGTCATCAACAAGC
hrdBrev	TGGACCTCGATGACCTTCTC
secDF-for	TCATTCTGGTCGGTGTGTTC
secDF-rev	GAGTCGTTCACCGAGTAGCC

The apramycin resistance gene of strain IB94 was removed by recombination between the FLP sites of the cassette, resulting in a 81 bp non-polar scar sequence (Gust et al., 2004). This apramycin-sensitive Δ*sco5282*::*scar* strain (IB95) was used in subsequent studies. The same procedure was followed to obtain strains lacking both genes for the two-component system, resulting in strains IB96 (Δ*sco5282-sco5283*::apra) and IB97 (Δ*sco5282-sco5283*::*scar*), using primers SCO5283For and SCO5282Rev.

### Genetic Complementation

Wild-type and mutant *sco5282* kinase genes were amplified by PCR with *Pfu* Ultra DNA polymerase (Agilent Technologies) from chromosomal DNA of M145 and 2L12, respectively, using primers clSCO5282For and clSCO5282Rev, which add an in-frame NdeI site at the start codon, and an EcoRI site at the 3′ end after the stop codon. The blunt-end PCR products were cloned in plasmid pBlueScript II SK (Alting-Mees and Short, 1989) digested with EcoRV, and their DNA sequence was confirmed using universal M13 primers. The wild type and mutant genes were then subcloned in vector pIJ6902 (Huang et al., 2005) using NdeI and EcoRI, thereby placing them under control of the inducible promoter *P*_tipA_. The resulting plasmids were transferred to *S. coelicolor* via conjugation with *E. coli* ET12567/pUZ8002. Sedimentation times of the mycelial pellets of the complemented strains were measured in the absence of thiostrepton.

### Merodiploid Strains

The ampicillin-resistance gene of cosmids StCB12 and StCB12*sco5282-D125G* were replaced by PCR targeting with the apramycin-resistance cassette from pIJ784 bearing the origin of transfer *oriT* (Gust et al., 2004), so that they could be introduced to *Streptomyces* by conjugation. Exconjugants for each cosmid were selected and maintained in the presence of both kanamycin and apramycin, to select for the presence of the integrated cosmids. Sedimentation times of the mycelial pellets grown in the presence of 25 μg/mL kanamycin were determined as described above.

### Phosphorylation Assays

The *sco5283* gene coding for the response regulator was amplified by PCR with primers clSCO5283for and clSCO5283rev (Table 1) and cloned in pET28a using the NdeI and EcoRI sites. The region encoding the cytosolic domain of the wild type and mutant sensor kinases (amino acids 80-375), were amplified by PCR from the wild type *sco5282* and mutant *sco5282-D125G* genes, respectively, with primers clSCO5282sol and clSCO5282rev (Table 1) and also cloned in pET28a with NdeI and EcoRI. The plasmids were then introduced into *E. coli* Rosetta 2 (Novagen) for protein expression. Protein purification was carried out using a Ni-NTA Superflow Cartridge (QIAGEN, 30721) in an FPLC system (ÄKTAprime plus; GE Healthcare) according to the manufacturer instructions.

Autophosphorylation and transphosphorylation assays were performed as described by Álvarez and Georgellis (2012).

### RNA Extraction, Sequencing and Analysis

For RNA extraction, three independent cultures of strains IB97 and M145-D125G were grown in 2xYT medium until mid-log phase (24 h), in shaking flasks without coils to allow pellet formation. Mycelium was harvested by filtration as described in Hopwood et al. (1985) and RNA isolation was carried out as described in Kieser et al. (2000), but using a modified Kirby mixture (1% SDS, 6% EDTA, 6% saturated phenol solution, 150 mM β-mercaptoethanol, 50 mM Tris–Hcl, pH = 8.3) as follows: pellets were filtered through ice-cold Whatman paper after pouring the cultures over a funnel containing ice, placed above the filter; the filter was washed with ice-cold water and the mycelium was then scraped into ice-cold modified Kirby solution and vortexed with an equal volume of 4 mm glass beads for 2 min, then five pulses of 30 s. The suspension was extracted with equal volumes of phenol:chloroform until the interphase was clear, then precipitated with sodium acetate and isopropanol. Total nucleic acids were treated with RNAse-free DNAse I (Ambion, AM2222; Thermo Fisher Scientific) until no PCR product was detected after 30 cycles using primers ATTB_FOR and ATTB_REV (Table 1). rRNA was depleted by subtractive hybridization with the Ribo-Zero Gram-positive kit (Illumina, MRZGP126); mRNA libraries for each of the three independent cultures of each strain were constructed using the TrueSeq stranded mRNA Library Prep kit (Illumina 20020594) and sequenced in an Illumina NextSeq 500 equipment, with up to 10^7^ paired reads/sample. Raw RNA sequencing reads are available through the NCBI Sequence Read Archive (BioProject Accession No. PRJNA499125).

Sequences were mapped to the reference genome of *S. coelicolor* A3(2) (NC_003888.3) and differential expression evaluated with packages DESeq2 1.18.1 (Love et al., 2014), edgeR 3.20.9 (Robinson et al., 2010) and NOISeq 2.22.1 (Tarazona et al., 2011). Genes were considered differentially expressed if they showed a log_2_ fold-change above 1 and an FDR-controlled *p*-value below 0.05. Genes that resulted positive with at least two tests were annotated with eggNOG-mapper (Huerta-Cepas et al., 2017).

### RT-qPCRs to Validate Differential Gene Expression

RNA obtained as described before was used in RT-qPCR using the Luna Universal One-Step RT-qPCR kit (NEB, E3005S), following the manufacturer’s instructions with 100 ng total RNA/reaction plus 5% DMSO (NEB, B0515). Differential expression was calculated according to the Δ⁢ΔCt method (Livak and Schmittgen, 2001), using the *hrdB* gene as calibrator. Primers used are listed in Table 1.

### Statistical Analysis

Pellet size and differential gene expression were compared by Student’s *t*-test. Due to the asymmetrical distribution of the sedimentation time, the corresponding assays were analyzed by the non-parametric Kruskal-Wallis test for multiple comparisons, with *post hoc* Dunn test with Benjamini-Hochberg correction of experiment-wise error rate. Statistical significance was determined at *p*-value < 0.05. All statistical procedures were performed using R version 3.4.4 (R Core Team, 2018).

## Results

### Experimental Evolution for Selection of Slow-Sedimenting Strains

Since wild type *S. coelicolor* grows in liquid medium as tight, dense mycelial pellets that settle quickly when the culture is left without shaking, we sought to isolate mutants showing dispersed growth by adapting a gravity-based sedimentation strategy used previously by Ratcliff et al. (2012). Our strategy consisted of sequentially transferring the uppermost part of settled cultures for reinoculation, as opposed to the strategy used to isolate multicellular derivatives of *S. cerevisiae*, which consisted of sequentially transferring the lower part of the settled cultures. Thus, any mutation which allowed for more dispersed mycelium and less dense mycelial pellets would be positively selected. By applying this selection to independent cultures of the wild type M145 strain, we observed more dispersed morphologies appearing after 12–56 transfers. The different isolates were transferred to solid medium and purified from single colonies after one sporulation round. Most of the purified strains no longer showed dispersed growth in liquid medium, or showed increased antibiotic production and reduced spore viability and were no longer considered. Only one strain, which was named 2L12, was phenotypically indistinguishable from M145 on solid MS medium and retained the slower sedimenting, dispersed growth phenotype when reinoculated in liquid medium.

**FIGURE 1:**
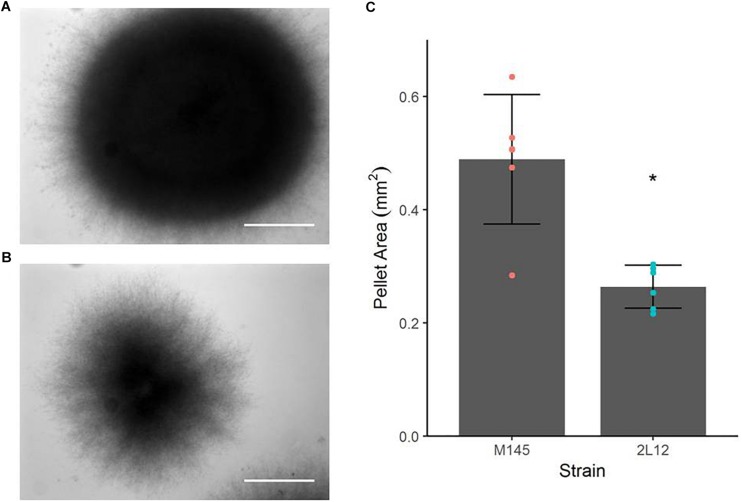
Pellet morphology of strains M145 and 2L12. Bright field microscopy of representative pellets, grown in 2xYT medium. **(A)** M145 pellet with an area of 0.621 mm^2^ and **(B)** 2L12 pellet with an area of 0.242 mm^2^. These areas are around the average of the distribution for each strain. It is worth noting that under these conditions, no pellets were found in the 2L12 strain of the size shown for M145, whereas in almost every experiment the M145 pellets reached at least this size. The scale bar corresponds to 0.2 mm. **(C)** Pellet area quantification. Each dot represents the average area of pellets from an independent culture; the raw data for each independent culture are shown in Supplementary Table S1. Bars represent mean ± standard deviation. An asterisk indicates significant difference at *p* < 0.01.

**FIGURE 2:**
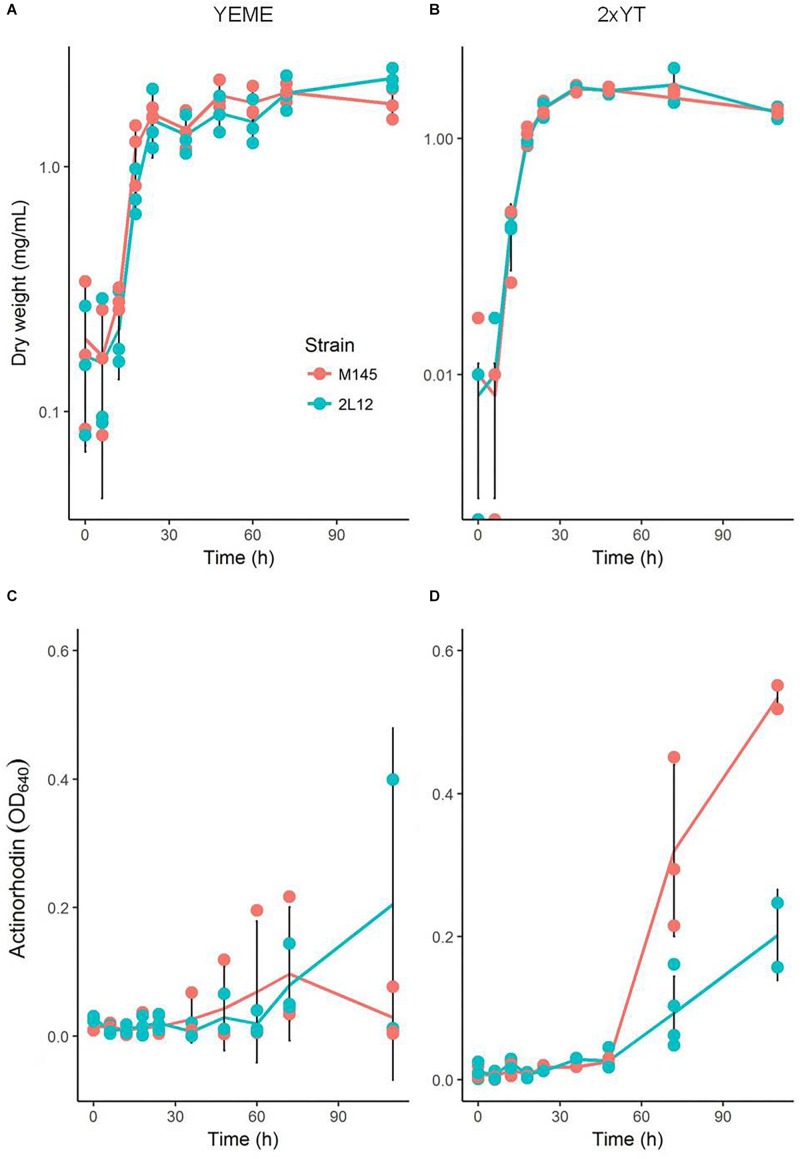
Growth and actinorhodin production by M145 and 2L12 strains. **(A,B)** show the growth curves in YEME and 2xYT media, respectively; **(C,D)** show actinorhodin production in YEME and 2xYT media. Each dot represents a measurement from each of three independent cultures; black vertical lines represent the means ± standard deviation.

Microscopically, the main difference between strain 2L12 and the parental strain M145 was the pellet size when both were grown in liquid media without sucrose (Figure 1). The pellets of 2L12 were smaller, like the one shown in Figure 1 (with an area of 0.242 mm^2^), and frequently appeared breaking apart, suggesting increased fragility. Pellets of the wild type M145 frequently reached at least the size of the pellet shown in Figure 1 (with an area of 0.621 mm^2^). It is worth noting that, under these conditions, we never found in the 2L12 strain a pellet of the size depicted for M145, whereas on almost every experiment the wild type pellets reached at least this size (Supplementary Table S1) The growth rate of 2L12 was indistinguishable from that of M145, either in liquid YEME medium with 34% sucrose or in 2xYT medium (Figures 2A,B), indicating that the reduced pellet size was not due to a major growth defect.

**FIGURE 3:**
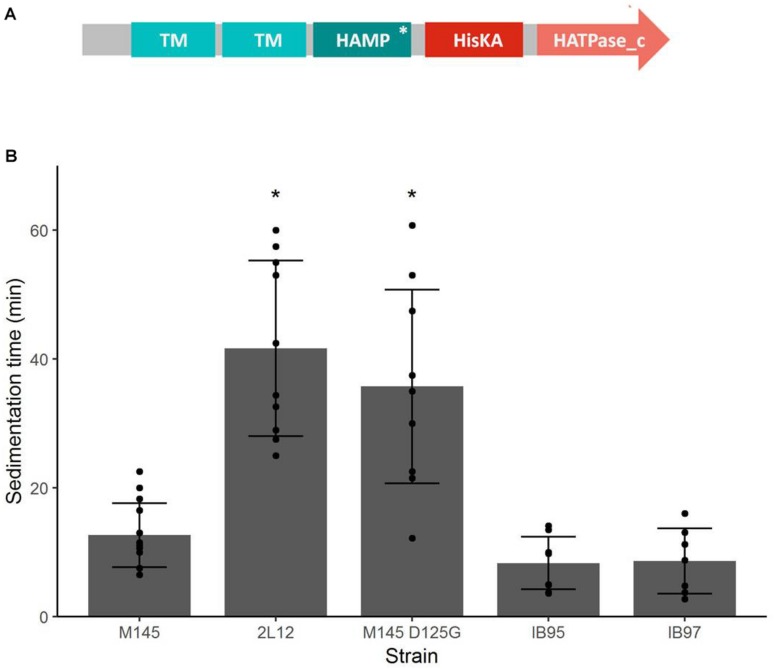
**(A)** Domain structure of the Sco5282 histidine kinase. TM, transmembrane domain; HAMP, HAMP domain; HisKA, histidine kinase domain, HATPase_c; ATPase domain. An asterisk shows the position of the D125G mutation. **(B)** Sedimentation time of *sco5282* mutants. Spores were germinated in viscous medium, then grown in thin medium. Pellets were deposited on top of a 2 cm tall 10% sucrose solution, and the time it took for the first pellet to reach the bottom of the tube was measured. Each dot represents the measurement from an independent culture; bars show the mean ± standard deviation; asterisks indicate statistical significance (*p* < 0.01).

In order to have an objective way to compare the phenotypes of the M145 and 2L12 strains, we measured the time it took for the pellets of either strain to settle through a 2 cm high column of a 10% sucrose solution in a 1 cm wide test tube, since loose and smaller pellets would take longer to settle to the bottom of the tube because of their lower density. As expected, the selected 2L12 strain settled significantly slower to the bottom of the tube than the M145 wild type strain (Figure 3), confirming the validity of our experimental evolution strategy (i.e., slower sedimentation due to increased mycelial dispersion).

We also measured total actinorhodin production of both strains grown in liquid media. In YEME, where neither strain forms large pellets due to the increased viscosity caused by the 34% sucrose present in the medium, there was no significant difference in actinorhodin production (Figure 2C). On the other hand, in 2xYT, where the wild type strain forms large, dense, pellets, only M145 produced actinorhodin (Figure 2D). This is consistent with previous studies which showed that most antibiotic production takes place inside pellets (Manteca et al., 2008). Together, these results demonstrate that, although strain 2L12 produces less actinorhodin under this condition, this difference is due to its morphology and is not caused by a mutation specifically affecting synthesis of this antibiotic.

### A Single Amino Acid Substitution Is Responsible for the Dispersed Growth Phenotype of Strain 2L12

In order to determine the genetic change responsible for the phenotype of 2L12, we sequenced the genome of this strain and also the genome of the isogenic M145 wild-type strain, grown from the same spore vial used for the evolution experiment that led to isolation of 2L12. After variant calling and filtering, a single nucleotide change, a T to C transition in nucleotide 5,754,703 of the *S. coelicolor* genome sequence (GenBank accession NC_003888) appeared as the likely mutation responsible for the differences observed between both strains (Supplementary Table S2). This mutation lies in the *sco5282* gene, which encodes a histidine kinase of a two-component system of unknown function. This kinase gene is translationally coupled to an upstream gene, *sco5283*, which encodes an OmpR-type response regulator. The Sco5282 kinase is predicted to be membrane-anchored by two transmembrane domains, followed by a cytosol located HAMP domain, which functions as a signal transmitter domain in many two-component sensor kinases (Parkinson, 2010). The HAMP domain is followed by the histidine kinase (HisKA) domain and a C-terminal HATPase domain (Figure 3A). The mutation found in 2L12 causes a substitution of an aspartate to a glycine (D125G) in the second helix of the HAMP domain of the kinase (herein referred to as *sco5282-D125G*). Analysis of the variants between genome sequences revealed two other possible mutations: one inside the *sco4820* gene and the other one in the intergenic region between *sco6571* and *sco6572*. However, these variants had much lower quality scores (179 and 160, vs. 222 of *sco5282-D125G*) and, in the case of the variant in the *sco4820* gene, not all the aligned reads showed the presence of this putative mutation, as opposed to *sco5282-D125G* where the mutation was present in all the aligned reads (Supplementary Table S2). For this reason, we focused on the mutation in the *sco5282* gene. Interestingly, when the presence of Sco5282/Sco5283 orthologs was analyzed by reciprocal BLASTP searches using the Actinoblast database (Chandra and Chater, 2014^2^) this two-component system was found to be prevalent in the *Streptomycineae*, but not in other actinomycetes suborders (Supplementary Figure S1).

**FIGURE 4:**
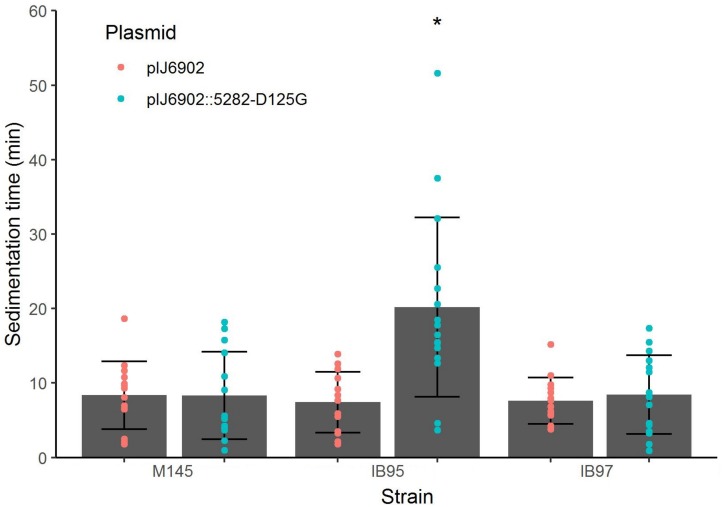
Sedimentation times of complemented strains. Vector pIJ6902 was introduced either empty or with the cloned *sco5282-D125G* gene into the indicated strains. Each dot represents the measurement from an independent culture; bars show the mean ± standard deviation; an asterisk indicates statistical significance (*p* < 0.01).

To demonstrate that the *sco5282-D125G* allele was responsible for the phenotype of strain 2L12, we transferred it to the wild type M145 background by recombining this mutation *in vivo*, using the StCB12 cosmid (which carries the *sco5282* and *sco5283* genes) as described in Materials and Methods. This resulting strain, M145*sco5282-D125G*, was grown in liquid culture and its sedimentation time was measured. It was found that this strain had the same slow-sedimentation phenotype as the 2L12 strain, demonstrating that the mutation in the histidine kinase gene *sco5282* is sufficient to cause a dispersed, slow-sedimenting morphology (Figure 3B). We also constructed a mutant carrying an in-frame deletion of the *sco5282* gene, IB95, and a mutant lacking both the kinase and response regulator genes (Δ*sco5282-5283*), IB97, and tested their sedimentation time. Interestingly, a wild-type phenotype, with fast sedimentation times was obtained for both the IB95 and IB97 mutant strains (Figure 3B). This result suggested that the *sco5282-D125G* allele is a gain-of-function mutation. It also implies that, during normal growth this two-component system is most likely inactive, since the absence of the Sco5282 kinase resulted in wild-type growth. To confirm that the *sco5282-D125G* allele is responsible for the dispersed morphology phenotype, we cloned the mutant allele under the control of the *P*_tipA_ promoter in the integrative expression vector pIJ6902, and introduced it into strain IB95, to complement the Δ*sco5282* null mutation. As can be seen in Figure 4, expression *in trans* of the mutant allele restored the dispersed growth phenotype, even without adding thiostrepton as inducer. This indicates that the mutant *sco5282-D125G* allele is able to produce a dispersed morphology even when it is expressed at the low constitutive level observed for the *P*_tipA_ promoter in the absence of thiostrepton (Ali et al., 2002). When the same plasmid was introduced into strain IB97, the mutant kinase alone did not cause the slow-sedimenting phenotype (Figure 4), indicating that the Sco5282-D125G mutant kinase exerts its effect through the Sco5283 cognate response regulator.

### The D125G Mutation Is Recessive

**FIGURE 5:**
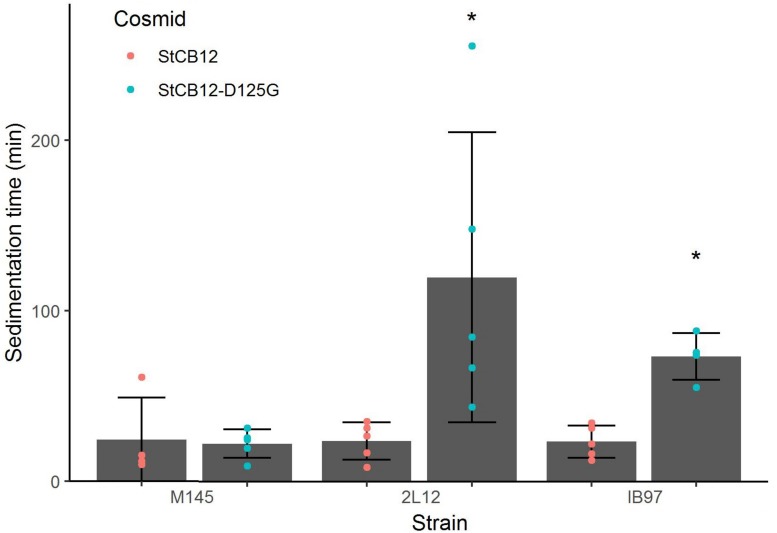
Sedimentation times of merodiploid strains. Merodiploid strains were constructed by insertion of the StCB12 cosmid harboring either a wild-type or mutant version of *sco5282-sco5283* as indicated. Each dot represents the measurement from an independent culture; bars show the mean ± standard deviation; asterisks indicate statistical significance (*p* < 0.05).

Since the *sco5282-D125G* allele appeared to be a gain of function mutation, we decided to determine whether it was dominant over the wild type gene. To this end, we introduced the pIJ6902 plasmid derivative carrying the mutant *sco5282-D125G* allele into the wild type M145 strain. Surprisingly, the mutant allele did not promote dispersed growth in the presence of the wild type gene (Figure 4) and therefore appeared to be recessive. To confirm this, we constructed merodiploid strains by inserting either the wild type StCB12 or the mutant StCB12*sco5282-D125G* cosmids into both the M145 and 2L12 strains, through homologous recombination. In this way, two copies of the two-component system, each one expressed from its own promoter, could be tested in the proper homozygous or heterozygous combinations. Measuring the sedimentation time of these strains revealed that insertion of a wild-type cosmid in strain 2L12 resulted in a wild-type phenotype, whereas insertion of the D125G cosmid did not result in a dispersed-growth phenotype in M145 (Figure 5). Both wild-type and mutant cosmids were also introduced into strain IB97, resulting in wild-type and mutant phenotypes, respectively (Figure 5). Taken together these results confirm that the *sco5282-D125G* mutation is recessive and also rule out the possibility that doubling the gene dose of the neighbor genes which are present in the cosmid might affect the growth phenotype.

### The D125G Substitution in the HAMP Domain Results in Activation of the Sco5282 Kinase

**FIGURE 6:**
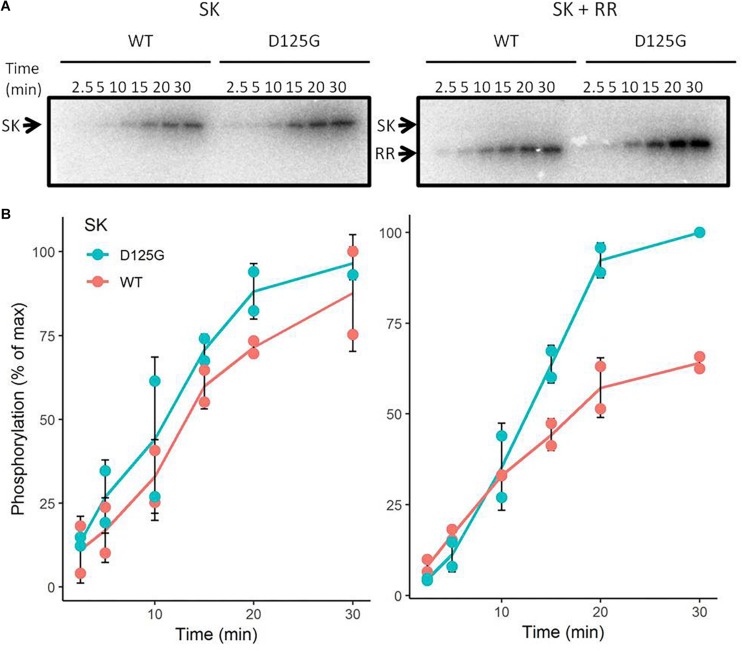
*In vitro* phosphorylation assays. Wild-type and mutant Sco5282 kinases were overexpressed in *E. coli*, purified and incubated with [γ-32P]ATP. **(A)** shows a time course of autophosphorylation in the absence of the Sco5283 response regulator and **(B)** shows a time course of transphosphorylation in its presence. Each dot represents an individual phosphorylation assay. The positions of the cytosolic portion of the Sco5282 histidine kinase and Sco5283 response regulator are indicated by SK and RR, respectively.

As mentioned above, the D^125^ to G mutation in Sco5282 appears to result in enhanced phosphorylation of the Sco5282/Sco5283 two-component system. To test this, His_6_-tagged versions of the Sco5283 protein and also of the cytosolic portions of the wild-type and mutant Sco5282 proteins were overexpressed in *E. coli*, purified by Ni-NTA-agarose affinity chromatography and used in *in vitro* autophosphorylation and transphosphorylation assays with [γ-^32^P]ATP (Figure 6). Although both the wild type and the mutant kinase proteins had similar kinetics regarding their autophosphorylation, the mutant Sco5282-D125G kinase exhibited a significantly higher transphosphorylation rate of the Sco5283 response regulator as compared to the wild type Sco5282 kinase protein. Net phosphorylation of Sco5282-D125G was also higher in the presence of Sco5283 as compared to Sco5282. Thus the D125G substitution in the Sco5282 kinase appears to be an activating mutation.

### Transcriptomic Differences Between Dispersed and Pelleting Strains

**FIGURE 7:**
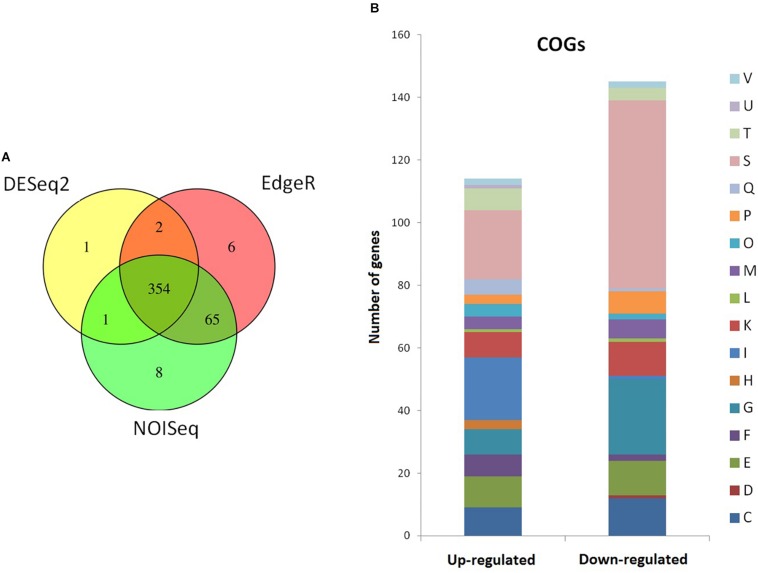
Summary of the differentially expressed genes. **(A)** Venn diagram showing the number of differentially expressed genes as analyzed by the statistical analysis packages DESeq2, EdgeR, and NOISeq. **(B)** Cluster of orthologous groups (COG) annotation of RNAseq-identified differentially expressed genes. C, energy production and conversion; D, cell cycle control and mitosis; E, amino acid metabolism and transport; F, nucleotide metabolism and transport; G, carbohydrate metabolism and transport; H, coenzyme metabolism; I, lipid metabolism; K, transcription; L, replication and repair; M, cell wall/membrane/envelop biogenesis; O, post-translational modification, protein turnover, chaperone functions; P, inorganic ion transport and metabolism; Q, secondary structure; T, signal transduction; U, intracellular trafficking and secretion; V, defense mechanisms; S, function unknown.

Since strain 2L12 is carrying an activating mutation in the Sco5282/Sco5283 two-component system, it is reasonable to argue that its dispersed growth is caused by the activation of genes that are normally not expressed or that have a low expression level. To investigate which genes were affected, we decided to analyze the transcriptomes of a strain with wild-type morphology and one with dispersed morphology. In order to maximize the differences in expression of target genes of the two-component system, we chose the strain M145*sco5282-D125G* as the strain exhibiting dispersed slow-sedimenting growth, and IB97 (i.e., the null Δ*sco5282-5283* mutant) as the tightly pelleting fast-sedimenting strain. Total RNA was extracted from three independent cultures of each strain at mid-log phase grown in 2xYT, the medium where morphological differences are maximal. After rRNA subtraction the samples were sequenced and gene abundances were compared using three statistical packages: DESeq2 (Love et al., 2014), edgeR (Robinson et al., 2010) and NOISeq (Tarazona et al., 2011). Comparisons were made between M145*sco5282-D125G* and IB97, so that positive log_2_ Fold Change represents genes that were either highly overexpressed in the dispersed strain, or repressed in the pelleting strain. 437 genes were found to be differentially expressed, and 354 of them were identified by all three statistical tests. Genes identified by at least two tests were annotated with the program eggNOG-mapper (Huerta-Cepas et al., 2017) to assign functional categories (Figure 7). Differential expression of relevant genes mentioned in the sections below and shown in Tables 2–4, was validated by qPCR as described in “Materials and Methods,” and shown in Table 5. A table showing the differentially expressed genes identified by all three statistical packages is provided as Supplementary Table S4.

**TABLE 2:** Differentially expressed genes involved in cell envelope maintenance.

				**FDR-controlled *p*-value**		
				
**Gene**	**Function**	**Log_2_ fold change**	**COG^1^**	**EdgeR**	**DESeq2**	**NOISeq**
SCO0753	Natural ribosomal product; predicted secretion signal peptide	6.3		1.5E-91	4.1E-15	1.0E-13
SCO0754	Secreted protein; homolog of type I secretion system protein HlyD	4.4	M	7.6E-51	1.5E-47	2.1E-14
SCO0755	ABC transporter component	4.25	V	2.6E-49	3.6E-60	2.9E-15
SCO0756	ABC transporter component	4.25	V	1.8E-50	1.1E-58	5.6E-16
SCO0757	Exopolyphosphatase; involved in phosphate metabolism	1.58	F, P	2.7E-08	7.8E-09	6.2E-06
SCO6160	*secDF*; alternative secretion system protein	2.89	U	1.4E-26	3.8E-02	0.0E+00
SCO6161	Secreted protein	1.51	–	1.4E-07	4.0E-06	2.8E-03
SCO6162	Response regulator	2.21	T	1.8E-16	5.3E-02	0.0E+00
SCO6163	Histidine kinase	2.13	T	1.7E-15	1.2E-01	0.0E+00
SCO1741	Secreted serine protease	4.10	O	5.3E-41	1.3E-36	1.1E-16
SCO0381	Polyprenyl glycosyl transferase; involved in exopolysaccharide syntesis	−1.86	M	3.3E-10	2.8E-01	1.5E-02
SCO0382	UDP-glucose/GDP-mannose dehydrogenase (*algD*); catalyzes the reaction GDP-mannose + NAD+ → GDP-mannuronate + NADH	−1.37	M	7.1E-06	3.7E-01	2.0E-02
SCO0836	Mechanosensitive ion channel	−1.31	M	1.2E-05	5.0E-09	1.6E-03
SCO2589	CDP-glycerol poly(glycerophosphate) glycerophosphotransferase; involved in teichoic acid biosynthesis	2.24	M	5.1E-17	7.6E-39	0.0E+00
SCO2590	CDP-glycerol poly(glycerophosphate) glycerophosphotransferase; involved in teichoic acid synthesis	2.37	M	7.6E-19	5.5E-41	3.3E-15
SCO2591	N-acetylmuramoyl-L-alanine amidase	−3.77	M	2.3E-41	3.0E-125	5.3E-14
SCO2962	Transferase (*matB)*; involved in exopolysaccharide synthesis	−1.73	M	3.0E-10	2.0E-23	1.1E-16
SCO6131	*D-alanyl-D-alanine carboxypeptidase*; penicillin-binding protein family 4	2.93	M	2.9E-27	5.9E-47	0.0E+00
SCO6164	*dksA*-like zinc-finger protein	2.53	K	5.7E-21	3.0E-01	0.0E+00
SCO6165	*dksA*-like protein	2.71	K	1.5E-23	1.7E-01	0.0E+00
SCO6166	*mreB/mbl2*; cell wall synthesis regulator	2.77	S	1.3E-24	9.1E-02	5.8E-04
SCO6167	Proline rich protein	1.48	S	5.6E-08	8.8E-05	5.6E-16

*^1^The cluster of orhologous groups (COG) categories in this table are: F, nucleotide metabolism and transport; K, transcription; M, cell wall/membrane/envelope biogenesis; O, post-translational modification, protein turnover, chaperone functions; P, inorganic ion transport and metabolism; T, signal transduction; U, intracellular trafficking and secretion; V, defense mechanisms; S, function unknown.*

**TABLE 3:** Differentially expressed genes for primary metabolism.

					**FDR-controlled *p*-value**		
					
**Gen**	**Function**	**Log_2_ fold change**	**Metabolic pathway**	**COG^1^**	**EdgeR**	**DESeq2**	**NOISeq**
SCO4979	Phosphoenolpyruvate carboxykinase	2.37	Gluconeogenesis	C	7.45E-19	3.81E-03	0.00E+00
SCO7040	Glyceraldehyde-3-phosphate dehydrogenase	1.48	Glycolysis/Gluconeogenesis	G	5.98E-08	1.18E-01	3.03E-05
SCO5047	Fructose-1,6-bisphosphatase	1.45	Gluconeogenesis	G	1.11E-07	3.97E-04	1.67E-15
SCO7638	Enolase	−1.22	Glycolysis/Gluconeogenesis	G	5.90E-05	2.76E-03	5.66E-03
SCO5423	Pyruvate kinase	−1.38	Glycolysis	G	7.14E-07	1.21E-04	2.89E-15
SCO1947	Glyceraldehyde-3-phosphate dehydrogenase	−1.67	Glycolysis/Gluconeogenesis	G	9.66E-10	8.63E-07	1.44E-15
SCO7511	Glyceraldehyde-3-phosphate dehydrogenase	−5.22	Glycolysis/Gluconeogenesis	G	2.49E-69	1.56E-09	1.01E-14
SCO6659	Phosphohexose isomerase	−4.00	Glycolysis/Gluconeogenesis	G	1.45E-45	1.41E-03	1.44E-15
SCO2774	Acyl-CoA dehydrogenase	1.56	Amino acid degradation	I	8.90E-09	1.41E-04	4.44E-16
SCO2776	Carboxylase	2.11	Amino acid degradation	I	3.14E-15	1.33E-01	3.77E-15
SCO2777	Carboxylase	2.12	Amino acid degradation	I	1.85E-15	1.16E-01	0.00E+00
SCO2778	Hydroxymethylglutaryl-CoA lyase	1.96	Amino acid degradation	I	2.54E-13	2.75E-01	1.06E-03
SCO2779	Acyl-CoA dehydrogenase	2.08	Amino acid degradation	I	6.17E-15	1.50E-01	0.00E+00
SCO4800	Isobutiryl-CoA mutase	1.16	Amino acid degradation	I	4.36E-05	2.09E-04	2.03E-03
SCO5398	Methylmalonyl-CoA epimerase	1.38	Amino acid degradation	I	5.03E-07	1.03E-05	0.00E+00
SCO5399	Acetyl-CoA acetyltransferase	1.63	Amino acid degradation	I	1.63E-09	1.41E-09	0.00E+00
SCO5415	Methylmalonyl-CoA mutasa	1.47	Amino acid degradation	I	7.03E-08	2.39E-01	0.00E+00
SCO6701	Acetyl-CoA acetyltransferase	1.44	Amino acid degradation	I	7.60E-07	4.84E-04	4.18E-03
SCO6702	Acetyl-CoA acetyltransferase subunit B	1.81	Amino acid degradation	I	3.26E-10	4.79E-02	8.24E-03
SCO4972	Xanthine dehydrogenase	2.65	Purine degradation	F	7.57E-23	1.38E-02	2.22E-15
SCO6209	OHCU decarboxylase	3.11	Purine degradation	F	8.62E-27	9.16E-19	5.50E-09
SCO6247	Dihydroorotase	2.92	Purine degradation	F	2.17E-26	7.02E-20	0.00E+00
SCO6248	Allantoate amidinohydrolase	2.43	Purine degradation	F	4.30E-19	2.53E-13	1.51E-11
SCO1679	Gluconokinase	−2.54	Pentose phosphate	G	5.38E-20	4.82E-02	6.66E-16
SCO6497	Transketolase	−1.99	Pentose phosphate	G	1.32E-12	3.37E-16	0.00E+00
SCO6658	6-phosphogluconate dehydrogenase	−3.92	Pentose phosphate	G	2.50E-44	1.07E-03	3.33E-16
SCO6661	glucose-6-phosphate 1-dehydrogenase	−4.84	Pentose phosphate	G	9.78E-62	1.86E-04	0.00E+00
SCO6662	Transaldolase	−5.33	Pentose phosphate	G	2.54E-71	1.10E-04	0.00E+00
SCO6663	Transketolase	−6.12	Pentose phosphate	G	1.51E-87	3.66E-05	4.33E-15
SCO1224	Ribose-5-phosphate isomerase	1.55	Pentose phosphate	G	1.33E-08	1.09E-06	0.00E+00

*^1^The cluster of orhologous groups (COG) categories in this table are: C, energy production and conversion; G, carbohydrate transport and metabolism; I, lipid transport and metabolism; F, nucleotide metabolism and transport.*

**TABLE 4:** Differentially expressed genes for transcriptional regulators.

				**FDR-controlled *p*-value**		
				
**Gene**	**Function**	**Log_2_ fold change**	**COG^1^**	**EdgeR**	**DESeq2**	**NOISeq**
SCO6164	*dksA*-like zinc-finger protein	2.53	K	5.72E-21	3.03E-01	0.00E+00
SCO6165	*dksA*-like protein	2.71	K	1.50E-23	1.67E-01	0.00E+00
SCO6166	*mreB/mbl2*; cell wall synthesis regulator	2.77	S	1.34E-24	9.08E-02	5.81E-04
SCO6167	Proline-rich protein	1.48	S	5.63E-08	8.79E-05	5.55E-16
SCO1897	DeoR-type regulator	2.15	K	3.20E-15	1.14E-06	2.21E-05
SCO0864	ECF sigma factor	1.69	K	1.27E-08	5.50E-05	7.98E-03
SCO4767	WhiD; sporulation regulator	1.45	K	7.60E-07	1.45E-04	7.85E-03
SCO6685	RamR; sporulation regulator	−1.48	T	7.25E-05	2.15E-02	1.33E-02
SCO3198	DeoR-type regulator	−1.59	K	1.13E-08	2.03E-01	9.10E-03
SCO6520	SigK; differentiation sigma factor	−1.65	K	4.32E-09	8.41E-07	4.09E-03
SCO0600	SigB; stress sigma factor	−1.70	K	4.36E-10	3.06E-23	3.22E-15
SCO7325	RsbV; SigB anti-anti-sigma factor	−1.28	T	7.68E-06	2.38E-03	4.55E-03
SCO4005	ECF sigma factor	−1.81	K	3.10E-11	4.31E-17	0.00E+00
SCO1658	GylR; glycerol responsive regulator	−2.05	K	2.88E-14	1.36E-07	1.78E-15
SCO2845	GntR-type regulator	−2.62	K	1.17E-16	2.93E-02	5.13E-03
SCO7314	SigM; differentiation sigma factor	−2.75	K	2.56E-22	7.35E-18	0.00E+00
SCO2846	ROK-type regulator	−3.49	K	3.83E-36	1.03E-02	4.44E-16

*^1^The cluster of orhologous groups (COG) categories in this table are: K, transcription; T, signal transduction; S, function unknown.*

**TABLE 5:** qRT-PCR validation of selected differentially expressed genes.

**Gene name**	**log_2_ fold change (M145-D125G vs. IB97)**^1^
*SCO0753*	8.10 ± 2.62^*^
*SCO0754*	5.79 ± 1.60^*^
*SCO6166 (mbl)*	5.27 ± 3.14^*^
*SCO7511 (gap3)*	−2.89 ± 1.81^*^
*SCO7040 (gap2)*	3.59 ± 2.58^*^
*SCO4979 (pckA)*	4.35 ± 2.99^*^
*SCO4767 (whiD)*	0.65 ± 2.73
*SCO2962 (matB)*	−0.86 ± 1.40
*SCO0600 (sigB)*	1.97 ± 3.76
*SCO6160 (secDF)*	3.68 ± 2.17^*^

*^1^Values represent the average of four biological replicates ± 95% confidence interval. An asterisk indicates statistical significance. Correlation between RNAseq and qRT-PCR resulted in *R*^2^ = 0.9129.*

### Genes Involved in Envelope Maintenance

Among the most overexpressed genes in the dispersed M145*sco5282-D125G* strain were many involved in protein secretion and envelope maintenance (Table 2). The single most overexpressed gene was *sco0753* which encodes a 77 amino acid peptide of unknown function. This gene is located upstream of an operon for a secretion system (*sco0754-sco0756*), homologous to a type I secretion system and similar to bacteriocin secretion systems (Håvarstein et al., 1995). The Sco0753 peptide has been predicted as a probable bacteriocin-like ribosomal lanthipeptide (van Keulen and Dyson, 2014). It is predicted to be very hydrophobic (57% of hydrophobic amino acids) reminiscent of the hydrophobic proteins that coat the hyphae during differentiation helping them to break free from the aqueous medium (Flärdh and Buttner, 2009). The fact that these four genes are conserved in many *Streptomyces* species, with the same genomic organization, suggests that this hydrophobic peptide is exported by the above mentioned secretion system.

Another gene with upregulated expression in the dispersed strain was *mbl2* (*sco6166*), which encodes one of the three MreB homologs present in *S. coelicolor*. Previous studies have shown that *mbl2* was expressed only in vegetative mycelium and repressed during differentiation (Heichlinger et al., 2011), in contrast to the other two paralogs, MreB and Mbl1, which were found to regulate cell wall deposition during sporogenic cell division, with a similar role to the one played by homologous proteins in unicellular bacteria.

The Sec system component SecDF (*sco6160*) was also greatly overexpressed in the dispersed strain. This protein consists of domains paralogous to SecD and SecF, but fused into a single polypeptide, which is normally expressed at very low levels (Zhou et al., 2014). It appears to be part of an operon which includes a two-component system, since all these genes had similar expression levels. SecDF promotes the release of secreting polypeptides, providing the proton motive force and enhancing protein secretion (Tsukazaki et al., 2011). This suggests that the dispersed strain is dealing with protein secretion stress, although it is not yet possible to tell whether this is a cause or a consequence of the mutant morphology.

On the other hand, exopolysaccharide synthesis proteins were repressed in the dispersed-growing strain. Of special interest is the gene encoding the MatB protein (*sco2962*), whose absence is known to lead to mycelial dispersion (van Dissel et al., 2015). However, the upstream gene, *matA*, was not differentially expressed. Further analysis is required to determine whether these genes are transcribed as a single unit, and in that case, the mechanism by which the system Sco5282-Sco5283 could be affecting such differential regulation. Even though *cslA* and *glxA* were not part of the set of genes identified by all three statistical analysis packages as differently expressed, their expression levels were lower in the dispersed mutant, and in the case of *cslA* this reduced expression was found to be statistically significant by two of the packages (see Supplementary Table S3).

### Primary Metabolism Genes

A majority of the genes identified as differentially expressed were annotated in the primary metabolism category. This was not surprising, since pellet formation is known to exert stress on the cells and limit the amount of oxygen and nutrients available to hyphae. Therefore, cell metabolism must adapt to the different conditions imposed by the morphology.

The main metabolic routes affected were those for branched-chain amino acids and purine degradation, which were stimulated in the dispersed strain, along with the regulatory enzymes of gluconeogenesis (Table 3). Glycolysis, on the contrary, was repressed in this strain. Together, these findings suggest that strain M145 *sco5282-D125G* is taking up carbon from amino acids and purines, the main components of 2xYT medium, while the pelleting IB97 strain is using carbohydrates. Most likely this is a consequence of the lysis occurring inside the pellets, which releases murein for the growing hyphae to feed on.

It is noteworthy that the genes for glyceraldehyde-3-phosphate dehydrogenase, for which *S. coelicolor* has three copies, were all differentially expressed. Two of them, including the most important *gap* gene during vegetative growth (*sco1947*, Li and Townsend, 2006) were repressed in the dispersed strain, while *sco7040* was overexpressed. This suggests specialized functions for the enzyme encoded by each gene, that may be related to their role in glycolysis and gluconeogenesis.

Other genes, strongly repressed in the dispersed strain, are those of the pentose-phosphate pathway.

### Regulatory Genes

The genes for 24 transcriptional regulators were found to be differentially expressed. Of those with known function, regulators of stress responses that indirectly promote differentiation and sporulation stand out. For example, SigB (*sco0600*) is an osmotic stress response sigma factor (Cho et al., 2001), which promotes differentiation by activating SigM (*sco7314*, Lee et al., 2005) and its own anti-anti-sigma factor RsbV (*sco4005*, Lee et al., 2004). Although SigK is a negative regulator of sporulation, its transcript is accumulated at the onset of the differentiation process (Mao et al., 2009). RamR is an orphan response regulator which also promotes sporulation indirectly (Keijser et al., 2002). All of these stress-response sporulation-promoting genes are overexpressed in the pelleting IB97 strain (Table 4), in agreement with previous knowledge that tight clumping induces stress in the mycelium.

No direct regulators of sporulation were identified, with the exception of WhiD, a regulator required for sporulation that normally is not expressed in liquid media, where no sporulation takes place (Molle et al., 2000). Nevertheless, this regulator was overexpressed in the dispersed mutant (Table 4), opposite to the trend of the indirect sporulation regulators. It has to be mentioned that little is known about the role of WhiD, other than the fact that it possesses a [4Fe-4S] nucleus capable of binding oxygen and nitric oxide (Jakimowicz et al., 2005, Crack et al., 2009).

Recently, it has been revealed that cytosolic copper is a major modulator of germination and development in *S. coelicolor*, and that its levels depend on the copper export system, encoded by the *sco2730* and *sco2731* genes. As is shown in Supplementary Table S3, no significant differences in the expression of these genes was observed in the dispersed mutant.

No regulators of antibiotic biosynthesis were differentially expressed, which is probably due to the fact that the RNA samples were taken at mid-log phase, when there is no antibiotic biosynthesis.

### Gas Vesicles

Among the most differentially expressed genes was one of the two clusters of gas vesicle genes in the *S. coelicolor* genome (genes *sco6499-sco6508*). Despite being widespread among actinobacteria, there are no reports of functional gas vesicles in these organisms (van Keulen et al., 2005). Transcriptional activation of this cluster has been seen in mutants of the arginine-synthesis regulator gene *argR* (Botas et al., 2018), of the nitrogen sensor *glnK* (Waldvogel et al., 2011), in response to saline stress, in a SigB dependent manner (Lee et al., 2005) and in the presence of antibiotics that target cell wall biosynthesis (Hesketh et al., 2011). These genes were greatly overexpressed in strain IB97, suggesting that their expression might be related to the general stress response shown by this strain.

## Discussion

In this study, we set up an experimental approach to focus on the factors governing mycelial aggregation in liquid culture. By working with liquid *S. coelicolor* cultures, it is possible to restrict the phenomenon to the non-sporulating vegetative mycelium. By applying a very general selective pressure, as is sedimentation speed, it was possible to isolate many different slow-sedimenting strains with dispersed morphology. Even though none of them reached a level of dispersion or fragmentation like the one seen in *S. venezuelae*, the fact that several different mutational events could be found, even in such a relatively short time (12 transfers), indicates that submerged culture morphology is a multifactorial trait that can be affected through many different ways. Initial concerns that too many mutations could arise in such experiments, complicating further analysis, were dissipated upon discovery that few generations were needed to obtain a visibly distinct phenotype, given that many different independent cultures were set up since the beginning. Strain 2L12 appeared on the 12th transfer, the fastest of all cultures used in this study, and it is most likely because of this that it differed from the wild type strain by a single point mutation.

It was important to identify mutations that did not have pleiotropic effects that could be causing fragmentation as a secondary effect to other major metabolic changes or that showed altered viability or differentiation. In this regard, we chose to work with strain 2L12 because it did not have any differentiation or sporulation problems. Even more so, growth kinetics showed that it was indistinguishable from wild-type M145, proving that it had no major defects in primary metabolism. It was surprising to find that in medium without sucrose, where morphological differences are the most pronounced between the two strains, growth proceeded at exactly the same rate, since pelleted forms are known to show reduced growth (Walisko et al., 2015). Nevertheless, it has been reported previously that those differences are especially seen in bioreactors, and not in cultures grown in shake flasks (van Dissel et al., 2015).

Metabolic adaptations were expected to take place in order to compensate for the different morphologies. Indeed, a third of the functional categories assigned to the differentially expressed genes belong to primary metabolism (Figure 7). Thus, the two different strains were on two different metabolic states despite growing at the same rate: the slow-sedimenting dispersed strain was using the carbon source in the medium through gluconeogenesis, whereas the fast-sedimenting tight-pelleting strain was using carbohydrates. This difference is probably due to the increased lysis that the pelleting strain is going through, since it has a larger proportion of hyphae inside the pellets, which is where lysis takes place (Manteca et al., 2008). Nonetheless, the pellets continue to grow (Manteca et al., 2008), which is the reason that they reach the same final biomass (Figure 2A).

### An Activating Mutation in the HAMP Domain

Genome sequencing revealed that strain 2L12 had a single point mutation that caused a substitution of an aspartate residue for a glycine in the HAMP domain of a histidine kinase of a two-component system of unknown function. Transferring the mutant allele to a wild-type background demonstrated that this mutation was sufficient to confer the dispersed slow-sedimenting phenotype. Further genetic analysis showed that this is not a loss-of-function mutation, that it does not change the specificity of the kinase for its cognate response regulator, and that it is a recessive allele. These are interesting findings for a HAMP domain mutation. The HAMP domain is composed of two helices, AS1 and AS2 that form a 4 helix-bundle when the kinase dimerizes. A phase-stutter at the contact of the helices allows them to rotate upon detection of the signal (Parkinson, 2010). Aspartate 125 is located in the membrane-distal part of the AS2 helix, opposite to the contact side of the helices, suggesting that it is probably not directly involved in the conformational changes of signal transmission.

Nevertheless, a substitution for a glycine is likely to interrupt the helical structure. Previously, destabilizing mutations in the membrane-distal half of the AS1 helix were identified as activating mutations of the PhoQ kinase (Matamouros et al., 2015). A model was proposed in which interactions between the HAMP and catalytic domains repress kinase activity. Extracellular signaling would disrupt these interactions, leading to kinase activation (Matamouros et al., 2015). Therefore, destabilizing mutations in the HAMP domain could lead to increased kinase activity. In this work, a similar mutation was found in the AS2 helix, with the same activating effect. Even though *in vitro* autophosphorylation was almost identical between the wild-type Sco5282 and Sco5282-D125G kinases, transphosphorylation of the response regulator by Sco5282-D125G was greatly increased, with a maximal phosphorylation level of the target almost twice that of the wild type. A possibility could be that the equilibrium between phosphorylation and dephosphorylation in the mutant kinase is displaced toward phosphorylation, resulting in increased activation *in vivo*.

This model could also explain why the activating D125G mutation is recessive. The HAMP domain is where the kinase dimerizes. In a heterozygous cell, both wild-type and mutant monomers are produced, which entails formation of heterodimers. These molecules would have one destabilized HAMP domain and one functional, which may still interact with the catalytic domain and repress it. Thus, heterodimers would display wild-type transphosphorylation activity, and consequently a wild-type phenotype.

### Transcriptional Changes Related to the Dispersed Morphology

*Streptomyces* morphology is a complex trait. Even though all species have a common life cycle in the soil, growing as a multicellular vegetative mycelium, their growth in the laboratory reveals widely different morphologies. *S. venezuelae* and *S. coelicolor* can form similar vegetative mycelia on solid medium, and yet they represent opposite sides of the morphology spectrum in liquid media: the first grows as fragmented, separate hyphae, while mycelium of the latter becomes inextricably matted in giant nets (van Dissel et al., 2014), which results in formation of large, dense pellets. Many parallels have been drawn between pelleted growth and biofilm formation: like a biofilm, a pellet’s matrix is composed of different polymers, at least extracellular DNA and polysaccharides (Xu et al., 2008). Polysaccharides are produced in germinating hyphal tips, to allow adhesion to the substrate and to each other (Zacchetti et al., 2016). Most surprisingly, when faced with nutrient scarcity, pellets disassemble, like a starving biofilm (Zacchetti et al., 2018).

Perhaps because most morphological studies of this genus have focused on sporulation, the part of the life cycle that occurs outside of the substrate, little is known about the importance of hyphal aggregation and biofilm-like mycelial growth. Some components of this extracellular matrix, however, are already known to be important in the *Streptomyces* life cycle: the polysaccharide synthesized by CslA is necessary to guide the hydrophobic chaplin layer that allows aerial hyphae to erect out of the water (de Jong et al., 2009). The other polysaccharide known to be a part of the *Streptomyces* extracellular matrix, synthesized by MatAB, seems to be non-essential for surface growth (van Dissel et al., 2015). Of course, nothing is known about the timing and regulation of these events.

The phenotype of strain 2L12 could be explained by the reduced expression observed in the *matB* gene, whose deletion is known to be sufficient to obtain dispersed growth. This is an important finding, since the Sco5282/Sco5283 two-component system would be the first identified regulator which affects expression of these exopolysaccharide synthases. Since *clsA* and *glxA* expression is also reduced, it is likely that reduced aggregation caused by lower expression of these genes also contributes to the phenotype. Even, though there is reduced expression of *matB*, the expression of the upstream *matA* gene is not altered, suggesting that changes in *matB* expression are most likely not caused by direct regulation by Sco5283. Therefore it appears it is more probable that reduced expression of *matB* is an indirect effect, probably exerted by one of the 24 transcriptional regulators identified as differentially expressed (Figure 7), 16 of which have no known targets. Further indication that *matAB* is not part of the direct regulon of Sco5282/Sco5283 is its narrow distribution among *Streptomyces* species: whereas *matAB* is lacking in some species like *S. venezuelae*, *sco5282-sco5283* has orthologs in all *Streptomyces* genomes present in StrepDB (Chandra and Chater, 2014).

Other genes identified as differentially expressed are more likely to be direct targets of the Sco5282-Sc05283 system. The lantibiotic-like peptide Sco0753 was the single most overexpressed gene in the strain carrying the activating Sco5282-D125G allele, with over 60-fold overexpression. Together with its putative secretion system encoded downstream, these genes were almost completely silent in the null mutant. *S. coelicolor* is known to produce another lantibiotic-like peptide with morphogenic properties, SapB, which is a surfactant peptide required for aerial mycelium erection in some conditions (Willey et al., 2006). It is possible that the Sco0753 peptide could induce changes in the surface of hyphae, although it is unlikely to function exactly as SapB, since it has no cysteins, which lantibiotics require to be processed (Sahl and Bierbaum, 1998).

Another morphogenic protein identified as differentially expressed was the MreB paralog Mbl2. Of the three paralogs encoded in the *S. coelicolor* genome, Mbl2 seems to be expressed exclusively in the vegetative mycelium and its deletion does not affect sporulation in solid medium (Heichlinger et al., 2011). Given the role of MreB coordinating synthesis of the cell wall, it is an obvious candidate to contribute to the alterations observed in strain 2L12.

Other genes differentially expressed suggest an activated protein secretion stress response. SecDF, which is normally not expressed, was activated in the presence of the constitutive kinase. It is interesting that a previous study considered the two-component system Sco5282-Sco5283 as a candidate homolog of *Bacillus subtilis* CssRS, which responds to the Sec secretion pathway stress. It was dismissed because its transcript levels remained unchanged after induction of protein secretion stress (Gullón et al., 2012), although that does not disprove the possibility that it may act as a sensor in this route. Sco5282 has only four amino acids in its extracellular loop, leaving the transmembrane helices as the only regions that could act as a sensor domain, for example to detect stalled Sec complexes. Curiously, among the overexpressed genes in our RNA-seq experiment, there was a serine protease, Sco1741, like the HtrA-like proteases of the CssRS response (Gullón et al., 2012). Protein secretion rate is bound to affect cell envelope structure, since it controls the rate at which remodeling enzymes can be translocated. Thus, strain 2L12 could have a weaker extracellular matrix due to improved secretion of polysaccharide-degrading enzymes.

Further research will be needed to discern the role of these proteins in vegetative mycelium morphology.

In general, our study provides insight into the molecular adaptations to morphological constraints. Most transcriptomic studies are performed in viscous media, where *Streptomyces* forms few or small pellets. In our experiment, RNA samples were specifically taken from 2xYT, a medium that promotes pellet formation, so that the main difference between strains was the pellet size. It is interesting that our results provide further confirmation that pelleted growth induces stress in the cells, as seen by the activation of many stress sigma factors in the null mutant. Surprisingly, gas vesicle genes were also activated in this condition. This cluster had been seen to be activated upon saline stress, in a SigB-dependent manner. SigB is also activated during pelleted growth, suggesting that it may also be regulating gas vesicle genes in this condition, which is unrelated to saline stress. A simple explanation could be that inner hyphae are lacking oxygen, and gas vesicles are formed in an attempt to find shallow waters with greater oxygen pressure. Another explanation is that gas vesicle proteins have a different role in a more generalized stress response, as has been previously suggested (Lee et al., 2005; Hesketh et al., 2011; Botas et al., 2018), in a process thus far unknown.

Even though this study has been carried out only with *S. coelicolor*, the prevalence of orthologs of the Sco5282/Sco5283 two-component system among different members of the *Streptomycineae* raises the possibility that genetic manipulation to activate this system could be used to modify morphology of *Streptomyces* species that grow in a pelleted form. It will also be interesting to explore whether the altered morphology can be used as a tool to increase extracellular enzyme production.

## Data Availability

The datasets generated for this study are available in the NCBI Sequence Read Archive accession number PRJNA499125.

## Author Contributions

EA-P, DG, GS-C, and LS-G designed the experiments. EA-P, GG-C, DG, and LS-G performed the experiments. EA-P carried out the statistical analysis. EA-P and LS-G wrote the first draft of the manuscript. All authors contributed to the manuscript revision, and read and approved the submitted version.

## Conflict of Interest Statement

The authors declare that the research was conducted in the absence of any commercial or financial relationships that could be construed as a potential conflict of interest.

## Footnotes

1 www.uusmd.unam.mx

2 http://streptomyces.org.uk/actinoblast
