# Supplementary material for: A Novel Two-Component System, Encoded by the sco5282/sco5283 Genes, Affects Streptomyces coelicolor Morphology in Liquid Culture
Source: Front Microbiol. 2019 Jul 9;10:1568. doi: 10.3389/fmicb.2019.01568 (PMC6629963; doi:10.3389/fmicb.2019.01568)
Supplement: Supplementary file 2 [file Table_2.DOCX]

Supplementary Table 2. Variant Calling for the Genome Sequence of Strain 2L12.

| **CHROMOSOME**  **POSITION** | **GENE ID** | **REFERENCE**  **SEQUENCE** | **ALTERNATE**  **SEQUENCE** | **QUALITY** | **% READS** | **SUBSTITUTION** |
| --- | --- | --- | --- | --- | --- | --- |
| 5251479 | SCO4820 | T | C | 179 | 50% (13/26) | T251A |
| 5754703 | SCO5282 | T | C | 222 | 100% (34/34) | D125G |
| 7275696 | SCO6571-SCO6572  (intercistronic) | GAAAAA | GAAAAAA | 160 | 100% (33/33) | None |
