# Supplementary material for: A Novel Two-Component System, Encoded by the sco5282/sco5283 Genes, Affects Streptomyces coelicolor Morphology in Liquid Culture
Source: Front Microbiol. 2019 Jul 9;10:1568. doi: 10.3389/fmicb.2019.01568 (PMC6629963; doi:10.3389/fmicb.2019.01568)
Supplement: Supplementary file 3 [file Table_3.DOCX]

Supplementary Table 3. Differential Expression of Selected Genes.

| Gene | Function | Log_2_ Fold Change | FDR-controlled p-value | | |
| --- | --- | --- | --- | --- | --- |
|  |  |  | EdgeR | DESeq2 | NOISeq |
| SCO2836 | *cslA* | -0.73 | 0.03 | **1.75E-05** | **0** |
| SCO2837 | *glxA* | -0.46 | 0.34 | 0.10 | **1.42E-05** |
| SCO2730 | Cu chaperone | 0.25 | 0.26 | 0.49 | 0.04 |
| SCO2731 | P-type ATPase | 0.19 | 0.84 | 0.61 | 0.05 |
