# Supplementary figures and images for: A Novel Two-Component System, Encoded by the sco5282/sco5283 Genes, Affects Streptomyces coelicolor Morphology in Liquid Culture
Source: Front Microbiol. 2019 Jul 9;10:1568. doi: 10.3389/fmicb.2019.01568 (PMC6629963; doi:10.3389/fmicb.2019.01568)

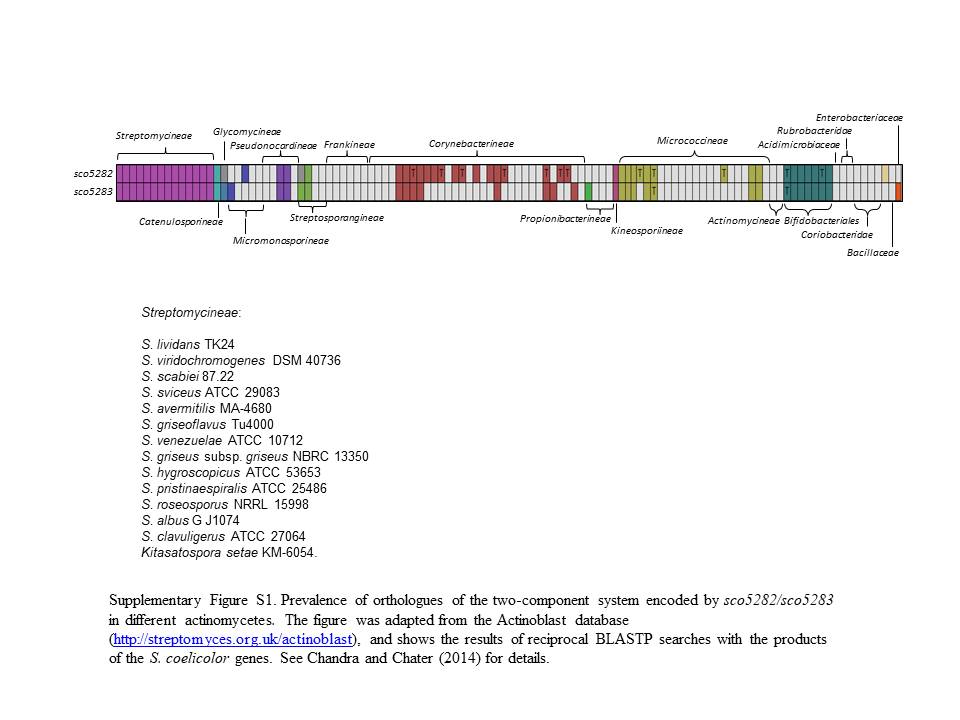

Supplement: Supplementary file 5 [file Image_1.JPEG]
